# Dosimetry, Efficacy, Safety, and Cost-Effectiveness of Proton Therapy for Non-Small Cell Lung Cancer

**DOI:** 10.3390/cancers13184545

**Published:** 2021-09-10

**Authors:** Bin Qiu, Yu Men, Junjie Wang, Zhouguang Hui

**Affiliations:** 1 Department of Radiation Oncology, Peking University Third Hospital, Beijing 100191, China; qiubin@pku.edu.cn (B.Q.); Junjiewang@pku.edu.cn (J.W.); 2 Department of Radiation Oncology, National Cancer Center/National Clinical Research Center for Cancer/Cancer Hospital, Chinese Academy of Medical Sciences and Peking Union Medical College, Beijing 100021, China; 3 Department of VIP Medical Services, National Cancer Center/National Clinical Research Center for Cancer/Cancer Hospital, Chinese Academy of Medical Sciences and Peking Union Medical College, Beijing 100021, China; sharon0303@126.com

**Keywords:** proton therapy, non-small cell lung cancer, radiotherapy

## Abstract

**Simple Summary:**

Non-small cell lung cancer (NSCLC) is the most common malignancy requiring radiotherapy (RT) as an important part of its multimodality treatment, the emergence of proton therapy may further allow for a sharper dose of build-up and drop-off com-pared to photon therapy, which has potentially improved clinical outcomes of NSCLC. Currently, there has been much emerging evidence focusing on dosimetry, efficacy, safety, and cost-effectiveness of proton therapy for non-small cell lung cancer (NSCLC) published, however, a comprehensive review comparing these therapies is, to date, lacking. This review focuses on these aspects of dosimetry, efficacy, safety, and cost-effectiveness of proton therapy for NSCLC.

**Abstract:**

Non-small cell lung cancer (NSCLC) is the most common malignancy which requires radiotherapy (RT) as an important part of its multimodality treatment. With the advent of the novel irradiation technique, the clinical outcome of NSCLC patients who receive RT has been dramatically improved. The emergence of proton therapy, which allows for a sharper dose of build-up and drop-off compared to photon therapy, has potentially improved clinical outcomes of NSCLC. Dosimetry studies have indicated that proton therapy can significantly reduce the doses for normal organs, especially the lung, heart, and esophagus while maintaining similar robust target volume coverage in both early and advanced NSCLC compared with photon therapy. However, to date, most studies have been single-arm and concluded no significant changes in the efficacy for early-stage NSCLC by proton therapy over stereotactic body radiation therapy (SBRT). The results of proton therapy for advanced NSCLC in these studies were promising, with improved clinical outcomes and reduced toxicities compared with historical photon therapy data. However, these studies were also mainly single-arm and lacked a direct comparison between the two therapies. Currently, there is much emerging evidence focusing on dosimetry, efficacy, safety, and cost-effectiveness of proton therapy for NSCLC that has been published, however, a comprehensive review comparing these therapies is, to date, lacking. Thus, this review focuses on these aspects of proton therapy for NSCLC.

## 1. Introduction

Lung cancer is the most commonly diagnosed malignancy and cause of cancer-related death, and patients affected by non-small cell lung cancer (NSCLC) comprise > 80% of the patients with lung cancer [1]. Radiotherapy (RT) is an important part of the multimodality treatment for NSCLC. With the advent of novel irradiation techniques, such as 3-dimensional conformal radiation therapy (3D-CRT), intensity-modulated RT (IMRT), and volumetric-modulated arc therapy (VMAT), the clinical outcome has dramatically improved with modern RT compared to conventional RT [2,3].

Nevertheless, the results of RTOG 0617 show that high prescription RT doses may be compromised in some situations, leading to serious toxicities, such as radiation-induced heart disease and, eventually, reduced survival rates due to the limited tolerance of the surrounding normal tissues (e.g., the lung, heart, and esophagus) [4]. Proton therapy is one of the types of RT that uses charged particles, allowing for a sharp dose build-up and drop-off compared to conventional photon therapy, which may further improve dose conformity, reducing damage to the surrounding normal tissue [3,5,6,7]. Thus, proton therapy is theoretically advantageous compared to conventional photon therapy [8]. 

During the past decades, proton therapy has been increasingly used worldwide, expanding the clinical trial portfolio rapidly [9]. Currently, emerging published studies have outlined the efficacy of proton therapy for NSCLC with a focus on dosimetry, efficacy, safety, and cost-effectiveness, however, a comprehensive review is lacking. This review summarized the published studies involving these aspects of proton therapy for NSCLC. The published studies were searched on PubMed using the keywords “proton therapy” and “lung cancer”. Eligible, studies were published between 1 April 1972 and 30 June 2021. Studies within these parameters that focused on dosimetry, efficacy, and safety, and cost-effectiveness were classified and included. 

## 2. Dosimetry 

Proton therapy has a completely different dose distribution compared with conventional photon beams. Unlike X-ray irradiation, the energy during proton therapy is deposited with depth and produces a maximum peak close to the end of the range [8]. The maximum peak is well known as the “Bragg peak”, which may be used for dose increment for cancer therapy while reducing the radiation dose to the normal tissue [10,11]. Indeed, published dosimetry studies have indicated that proton therapy significantly reduces the dose to normal structures, especially in relation to the lung, heart, and esophagus, when maintaining similar robust target volume coverage to the clinical target volume (CTV) in both early and advanced NSCLC compared with photon therapy. Currently, passive scattered proton therapy (PSPT) and active pencil beam scanning (PBS) are the two forms of proton therapy in use [12]. The former form uses one or two levels of scatterer to widen the proton beam enough in order to cover the target, while the latter form uses magnets to deflect the proton beams directly, rather than a scatterer. The majority of comparative studies about dosimetry included patients with advanced NSCLC. Studies on the impacts of proton therapy on early-stage cancers were limited, as listed in Table 1. Those that do exist were mainly conducted in a retrospective manner, and include only two prospective studies [13,14,15,16,17,18,19,20,21,22,23,24,25,26,27]. 

### 2.1. PSPT

Among the limited studies using proton therapy for early-stage NSCLC, PSPT has favorable CTV coverage and distributes lower mean doses to the normal tissues, compared with photon therapy. As reported by Wink et al. [15] in a retrospective study including 25 patients, CTV doses were more homogenous, and the dose directed to the spinal cord was lowest with PSPT, compared with IMRT, VMAT, and CyberKnife. Wang et al. [13] reported that in 24 patients with stage I NSCLC, the 95% isodose line of PSPT covered more CTV than that of 3D-CRT (86.4% versus 43.2%), and the mean dose to lung, heart, esophagus, and spinal cord was also lower, as well as V_5Gy_ and V_20Gy_ to the lungs. The two studies mentioned above were focused on early-stage patients undergoing a hypo-fractionated radiation therapy regimen (60–66 Gy in 8–10 fractions). 

For locally advanced NSCLC, PSPT also reduces the dose to the critical normal tissues and prevent lower-dose target coverage. One of the only two prospective studies indicated that PSPT could keep the dose to the target at 70 Gy for patients with stage IA–IIIB NSCLC, while sparing the lung, compared with 3D-CRT/IMRT (mean lung dose, 13.5 Gy versus 18.9 Gy/16.4 Gy) [17]. The second prospective study was a phase III trial, reported by Giaddui T et al. comparing the dose parameters for 26 lung IMRT, with 26 proton PSPT plans. As a result, the dose parameters for the IMRT and PSPT plans were very close. However, the PSPT plans led to lower dose values for normal structures (including lung V_5Gy_, 34.4% versus 47.2%; maximum spinal cord dose, 31.7 Gy versus 43.5 Gy; heart V_5Gy_, 19% versus 47%; and heart V_30Gy_, 11% versus 19%) [23]. The dosimetry comparative studies of PSPT for advanced-stage patients were mostly using conventional regimens (66–74 Gy in 33–37 fractions).

However, two respective comparative studies revealed similar or worse dose distribution to the lung or esophagus for PSPT. Wu et al. [22] reported that in 33 patients with stage III NSCLC, all of the dose parameters of proton therapy were lower than 3D-CRT, except for the esophageal dose, which was slightly higher than that of the photon plan (V_50Gy_, 20.2 versus 16.6%), but the difference was not significant. Another study by Shusharina et al. [24] with 83 patients (II-IV stage NSCLC), reported that, although higher lung V_5Gy_ was observed for IMRT, whereas higher V_60Gy_ for was observed for PSPT, the mean lung dose was similar. However, these two studies were both retrospectives and may have been prone to selection bias.

### 2.2. PBS

PBS may have advantages compared with PSPT in terms of offering greater dose conformality [28]. The entry dose of PSPT is often unmodulated, even after using the layer-stacking method [5]. Meanwhile, the movement of the target during PSPT causes dose distribution disturbances due to interplay and blurring effects, which leads to dose misses and unwanted doses to healthy organs. PBS generates more conformal high-dose volumes than PSPT, with significant sparing of nearby organs, and intensity-modulated proton therapy (IMPT) can be comprehended [29]. Gjyshi et al. [30] compared two independent cohorts with locally advanced NSCLC (86 received PSPT and 53 received IMPT) with data extracted from a prospective registry study, and found that lower mean radiation doses to the lungs (16.0 Gy versus 13.0 Gy, *p* < 0.001), heart (10.7 Gy versus 6.6 Gy, *p* = 0.004), and esophagus (27.4 Gy versus 21.8 Gy, *p* = 0.005) resulted in lower rates of pulmonary (28% versus 3%, *p* = 0.006) and cardiac (14% versus 0%, *p* = 0.05) toxicities for IMPT.

IMPT is also sensitive to uncertainties or target motion. Four-dimensional (4D)-computed tomography (CT) ventilation imaging-guided proton therapy, based on breathing patterns, may be helpful for reducing uncertainties and dosing to the normal tissues [31,32,33]. IMPT via a deep-inspiration breath-hold, deformable image registration with daily adaptive proton therapy, and liver-ultrasound-based motion modeling may also provide additional benefits [34,35,36,37]. FLASH proton therapy which optimizes tissue-receiving dose rate distribution and dose distribution may also provide substantial improvements, compared to IMPT, for normal tissue sparing [38].

As displayed in Table 1, published dosimetry comparative studies with proton and photon therapy for IMPT were all retrospective studies with <30 cases. The only study for early-stage NSCLC (15 patients with centrally/superiorly located stage I NSCLC) was reported by Register et al., which revealed that IMPT and PSPT significantly reduced doses to the surrounding normal tissues while maintaining a high radiation dose focused on the tumor, compared with SBRT (total lung volume receiving 5 Gy, 10 Gy, and 20 Gy, respectively) [14]. The rest of the dosimetry studies included patients with stage III NSCLC, and consistent results were observed for IMPT with comparable, if not better, CTV dose homogeneity/coverage while sparing the lung, heart, spinal cord, and esophagus to a greater extent. In addition, IMPT allowed for further dose escalation, compared with photon therapy [16]. Zhang X et al. reported that IMPT might allow further dose escalation (a mean maximum tolerated dose to 83.5 Gy or 84.4 Gy) and prevent lower-dose target coverage for the treatment of stage IIIB NSCLC, while sparing more lung, heart, spinal cord, and esophagus, compared with IMRT, and with similar normal tissue sparing compared with PSPT [16]. Therefore, PBS, which is gradually replacing PSPT in the clinical practice of proton therapy, may potentially overcome the limitations of PSPT and reduce treatment-related toxicity. 

Notably, some studies reported special characters for proton, compared with photon therapy. Palma G et al. [39] reported that in 178 patients with advanced NSCLC who were treated with PSPT/IMRT (66/74 Gy, conventional fractionation) with concurrent chemotherapy, significant dose differences of the heart and the lower lungs was found in the 40 patients who developed clinically symptomatic pneumonitis, compared with those without pneumonitis, which may substantiate potential factors in the development of pneumonitis. Harris et al. [40] retrospectively reported that in 160 (78 photons, 82 protons) patients with locally advanced NSCLC who were treated with chemoradiotherapy, among them, 40 (20 photons, 20 protons) patients exhibited grade ≥2 pneumonitis. After multivariate analysis, V_40Gy_ turns out to be statistically significant for proton and a potential pneumonitis predictor is V_40Gy_ ≤ 23%, and not V_20Gy_ or Dmean which are traditionally used in photon therapy. However, the dose-response of proton therapy for normal tissue complications has been validated as similar to that of photon therapy, based on a pneumonitis model [41]. Xiang et al. [42] identified 450,373 pediatric and adult patients with cancers (33.5% with 3D-CRT, 65.2% with IMRT, and 1.3% received proton therapy) from the National Cancer Database, and during a median follow-up of 5.1 years, the rate of diagnosed secondary cancer was 1.55% per year, suggesting that proton therapy was associated with lower risk of secondary cancer compared with IMRT (adjusted odds ratio 0.31, *p* < 0.0001). Further study with a long follow-up duration is needed.

In summary, proton therapy (both PSPT and PBS) has a dosimetry advantage compared with photon therapy both for early-stage or advanced NSCLC, both theoretically and clinically. This advantage leads to favorable or comparable CTV coverage with more homogenous dose distribution and more normal tissue sparing in most of the studies, and potentially with improved clinical outcomes involving efficacy and safety, which are then discussed below.

## 3. Efficacy and Safety

The clinical outcomes of proton therapy varied from study to study. Previously published prospective studies and nationwide retrospective studies involving proton therapy for NSCLC, with reported local control rate/failure rate/overall survival (OS)/progression-free survival (PFS)/disease-free survival (DFS), are included and summarized in Table 2 for early-stage NSCLC [43,44,45,46,47,48] and in Table 3 for advanced-stage NSCLC [49,50,51,52,53,54,55,56]. For early-stage NSCLC, six prospective studies and one nationwide retrospective study were found. In addition, seven prospective studies and one nationwide retrospective study were found for advanced-stage NSCLC. Notably, the proton therapy was delivered via the form of PSPT or IMPT, and some studies were using both or were not indicated; for this case we only used “proton therapy” in the tables and the following context.

### 3.1. Early-Stage NSCLC

In a systematic literature review published for proton therapy treating early-stage lung cancer, including one phase II study, two prospective studies, and two retrospective studies published before the 2010s, the 2-year local control rate, OS, and cause-specific survival rates were 87%, 31–74%, and 58–86%, respectively [10]. In addition, the 5-year local control rate, OS, and cause-specific survival rates were 57%, 23%, and 46%, respectively [10]. As revealed by the studies listed in Table 2, including more recent studies, the 2-year local control rate was reported with 87–95%, and the 2-year OS and cause-specific survival rate were 42.9–74% and 86%, respectively. Pneumonitis was the major toxicity, while therapy-related toxicities of grade >3 were not common. 

In particular, the largest prospective study was focused on hypo-fractionated proton therapy for 111 patients with stage I NSCLC, as accounted by Bush et al., where the clinical outcomes of the entire group improved as the prescription dose increased with a 4-year OS of 18% (for 51 Gy), 32% (for 60 Gy), and 51% (for 70 Gy). The rest of the prospective studies included a relatively small sample size (ranging from 21 to 43 patients). The studies by Iwata et al. (46) included 43 patients receiving proton therapy and 27 patients receiving carbon-ion therapy. For all of the 70 patients, the 4-year OS, local control, and PFS rates were 58%, 75%, and 46%, respectively, with no significant differences between the two regimens. Grade 3 pulmonary toxicity was observed in two patients [46]. The only nationwide retrospective study of PSPT was reported by Ohnishi et al. [48], which is the largest study, including 669 patients with stage I NSCLC. The median follow-up period was 38.2 months for all patients. The 3-year OS and PFS rates were 79.5% and 64.1%, respectively. The incidence of grade ≥ 3 pneumonitis and dermatitis were 1.7% and 0.4%, respectively. In addition, photon therapy may also be used in special circumstances. Kim et al. [58] retrospectively reviewed 30 patients suffering from complications, with stage I-II NSCLC and idiopathic pulmonary fibrosis (22 patients managed with X-ray and eight patients with proton therapy). During the follow-up (median 11 months), four patients who died within one month of the onset of pulmonary symptoms were all treated with X-ray. The 1-year OS was 46.4% for X-ray and 66.7% for proton therapy (*p* = 0.081). Nagata et al. [59] reported proton therapy (66 Gy in 10 fractions) for 48 patients with stage I ground-glass opacity (GGO)-type lung cancer, the 3-year OS, DFS, and local control were 91.7%, 85.4%, and 92.5%, respectively. Radiation pneumonitis was frequent (89.6%), followed by rib fracture and cough (both 27.1%) while all the complications were grade ≤ 2. 

A direct comparative prospective study of proton therapy and photon therapy is now lacking for early-stage NSCLC. Li et al. [60] compare lung changes in patients with early-stage NSCLC after matching 23 pairs of stereotactic body radiation therapy with protons (SBPT)/SBRT, including five patients treated with both modalities. Normal lung responses following SBPT significantly increased in the early time (<6 months, median 3 months), and did not then change significantly thereafter; dose-defined lung inflammation occurred earlier compared with SBRT, while no significant difference in the maximum response was reported. These differences were the most pronounced in insensitive (response > 6 HU/Gy) patients. In a meta-analysis published in 2010 [3], which included five studies on proton therapy, the 5-year OS for proton therapy was 40%, which was significantly higher than conventional RT (20%) and similar to that for SBRT (42%) in stage I inoperable NSCLC. Proton therapy resulted in no grade 3/4 esophagitis, dyspnea, or treatment-related deaths [3]. Only four out of 336 patients had grade 3/4 pneumonitis [3]. Moreover, another meta-analysis published in 2017, which included 72 SBRT studies and nine hypo-fractionated proton studies (mostly single-arm) for the treatment of early-stage lung cancer. Proton therapy was associated with improved OS and PFS in univariate meta-analysis. However, the OS benefit did not reach statistical significance after multivariate meta-analysis, but the 3-year local control still favored proton therapy [5]. All of the studies included in the above two meta-analyses had a small sample size and were single-arm without direct comparison; the comparison being based on historical data. Therefore, the conclusion regarding the efficacy and safety of proton therapy over photon therapy should be further explored in prospective comparative studies.

### 3.2. Locally Advanced NSCLC

Studies with proton therapy for locally advanced NSCLC are limited. Most of these studies are single-armed, and studies presenting a direct comparison with photon therapy are also limited, as shown in Table 3. In the only randomized controlled trial (RCT) which compared IMRT (*n* = 92) with PSPT (*n* = 57), both with concurrent chemotherapy, PSPT resulted in less lung dose, of 5 to 10 Gy, while exposing more lung to ≥20 Gy. The heart was less exposed to all dose levels (5 to 80 Gy). The grade ≥ 3 radiation pneumonitis rate was 8.1% (IMRT, 6.5% versus PSPT, 10.5%) and corresponding local failure rates were 10.7% (IMRT, 10.9% versus PSPT, 10.5%) [53]. The historical data comparison in MD Anderson Cancer Center by Sejpal et al. [49], included 62 patients treated with chemotherapy and proton therapy (period 2006–2008), 74 patients with chemotherapy and 3D-CRT (period 2001–2003), and 66 patients with chemotherapy and IMRT (period 2003–2005). The median follow-up times were 15.2, 17.9, and 17.4 months, respectively. As a result, the rates of grade ≥ 3 pneumonitis and esophagitis were significantly lower (proton, 2%, and 5%; 3D-CRT, 30%, and 18%; IMRT, 9%, and 44%), despite the higher radiation dose in the proton group (74 Gy versus 63 Gy in the other groups). Kim et al. [61] retrospectively reviewed 223 patients with locally advanced NSCLC who received concurrent chemoradiotherapy (29 with PSPT and 194 with IMRT), and found that the lung V_≥5–20Gy_ and the mean dose were significantly lower in patients receiving PSPT than in those receiving IMRT (*p* < 0.001). Severe radiation-induced lymphopenia was associated with lung V_5Gy_ and worse 2-year OS, which still favors PSPT (odds ratio 0.13, *p* = 0.003). In another study by Kim et al. [62], 25 patients underwent PSPT and 194 patients underwent IMRT, and those patients undergoing PSPT exhibited less radiation exposure in the lung, heart, and spinal cord compared to IMRT. The 2-year locoregional control rates (IMRT 72.1% vs. PSPT 84.1%; *p* = 0.287), the rates of esophagitis (grade ≥ 3) (IMRT 8.2% vs. PSPT 20.0%; *p* = 0.073), and rates of radiation pneumonitis (grade ≥ 2) (IMRT 28.9% vs. PSPT 16.0%; *p* = 0.263) were all similar, although worse pulmonary function at the baseline was reported for patients receiving PSPT. The largest retrospective study of the National Cancer Database included patients with stage II and III NSCLC (photon 1549 and proton 309, after propensity-matched analysis), and here, proton therapy was associated with improved 5-year OS (22% versus 16%, *p* = 0.025) compared with photon radiotherapy [52].

Concurrent radio-chemotherapy with proton therapy is currently undergoing testing in a prospective manner for locally advanced NSCLC. The largest prospective study, by Nguyen et al. [50] included 21 patients with stages II and 113 patients with stage III NSCLC, who were treated with PSPT and chemotherapy. The median OS was 40.4 months and 30.4 months, respectively, with a corresponding 5-year DFS of 17.3% and 18.0%. One patient with esophagitis (grade 4) and 16 patients with grade 3 complications (pneumonitides in two cases, esophagitis in six cases, and dermatitides in eight cases) was noted. In an open-label, single-group phase 2 trial [55], 64 NSCLC patients (stage IIIA, 30; IIIB, 34) were enrolled and treated with concurrent chemotherapy (carboplatin-paclitaxel) and high dose PSPT (74 Gy). The median OS was 26.5 months (5-year OS, 29%) and 5-year PFS was 22%. The outcomes seem superior to previously published breakthrough results with photon RT for locally advanced NSCLC (median OS 16–17 months), but are comparable with the control arm of the recently updated PACIFIC trial [63,64,65]. The control arm in the PACIFIC trial had a median OS of 29.1 months, and a 5-year OS/PFS of 33.4%/19 %. However, patients with tumor progression or with grade 2 or higher pneumonitis were excluded from the study [65]. Distant failures (48%) were the main causes, compared with local (16%) and regional (8%) recurrence [55]. Acute toxic effects were all ≤ grade 3 (acute esophagitis in 36% and pneumonitis in 2% of patients). Late toxic effects were also recorded (including 2% grade 2 esophageal stricture, 2% grade 4 esophagitis, 28% ≤ grades 3 pneumonitis, 3% bronchial stricture, and 2% grade 4 bronchial fistula). There were no acute or late grade 5 toxic effects [55]. 

The prospective investigation of a radical regimen with hypo-fractionated or dose-escalation for proton therapy turned out to be well tolerated. Hoppe et al. [56] reported a multicenter phase I trial that enrolled 18 patients with stage II/III NSCLC, although the study closed early because of slow accrual and competing enrollment with no maximum tolerated dose identified. Proton therapy delivered at 2.5 Gy per fraction in five patients, 3 Gy per fraction in five patients, 3.53 Gy per fraction in seven patients, and 4 Gy per fraction in 1 patient to a total dose of 60 Gy resulted in only 2 severe adverse events attributed to chemotherapy occurred among seven patients treated at 3.53 Gy per fraction. The results indicated that hypo-fractionated proton therapy, combined with concurrent chemotherapy, has an acceptable toxicity profile. Hoppe et al. [51] reported a phase II study for dose-escalated proton therapy with concurrent chemotherapy, 14 patients with 9 stage IIIA and 5 stages IIIB NSCLC were included, and the dose-escalation of 74 to 80 Gy was also well-tolerated. The median OS was 33 months with a median PFS of 14 months. The 2-year OS rate was 57% and the 2-year PFS rate was 25%. Late grade 3 gastrointestinal and pulmonary toxicity was noted in one patient, while no grade 3 acute toxicities related to proton therapy. In addition, Ohnishi et al. [66] retrospectively reported that in 45 patients with stage III NSCLC managed with PSPT (74 Gy, concurrent chemotherapy), the 3-year and 5-year OS/PFS rates were reported with 63.7%/22.2% and 38.8%/17.7%, respectively, with a median of 49.1/13.1 months. No grade 4/5 acute/late non-hematologic toxicities were observed.

In addition, proton therapy was tested in a special setting for locally advanced NSCLC, such as salvage, postoperative, or palliative manners. Shin et al. [67] retrospectively reviewed 53 patients who received salvage proton therapy for locoregionally recurrent NSCLC, and found that the median disease-free interval was 14 months. The 2-year OS rate, local control rate, and PFS rate were 79.2%, 68.2%, and 37.1%, respectively. Eight patients (15.1%) with grade 3 toxicities occurred (two esophagitis, three dermatitis, and four pulmonary toxicities). Postoperative radiation therapy for 136 patients (61 proton therapy, 75 IMRT) was reported by Boyce-Fappiano et al. [68], and it was found that the organ-at-risk (OAR) was more spared with proton therapy compared with IMRT, including the heart (mean 2.0 vs. 7.4 Gy, *p* < 0.01; V_30Gy_ 2.6% vs. 10.7%, *p* < 0.01) and lung (mean 7.9 vs. 10.4 Gy; *p* = 0.042; V_5Gy_ 23.4% vs. 42.1%, *p* < 0.01; V_10Gy_ 20.4% vs. 29.6%, *p* < 0.01). The total toxicity was also significantly reduced (OR, 0.35; *p* = 0.017), including cardiac toxicity (14.7% for IMRT vs. 4.9% for proton therapy, *p* = 1.09) and grade ≥ 2 pneumonitis (17.0% for IMRT and 4.9% for proton therapy, *p* = 0.104). Iwata et al. [69] retrospectively studied proton therapy with concurrent chemotherapy and respiratory-gated, image-guided techniques, and adaptive planning, for unresectable stage III NSCLC (47 patients), the 2-year OS/local control rate/PFS were 77%/59%/84% and 5-year OS/local control rate/PFS were 61%/43%/37%, respectively. No ≥ grade 3 pneumonitis and deterioration of quality of life were observed.

In summary, photon therapy was promising for locally advanced NSCLC with improved clinical outcomes and reduced toxicity when compared with historical photon therapy data, although the direct comparison was limited. Toxicities were acceptable, and pneumonitis/esophagitis were the most common observed toxicities. Rates of severe (grade 3) toxicities of proton therapy were lower than in photon therapy in the retrospective study, but this was not the case in the only RCT [53]. Further exploration of concurrent radio-chemotherapy/hypo-fractionated or dose-escalation regimen in the setting of PBS is ongoing (such as the ongoing RCT: RTOG 13-08) [23], and direct comparison is warranted.

## 4. Cost-Effectiveness

Currently, proton therapy is being used as a treatment for various cancers [70]. However, owing to low cost-effectiveness, the necessity of proton therapy was discussed in several studies [70]. Compared with photon therapy, the initial cost was 2.4-fold higher for proton therapy, however, after adding the costs of treating adverse effects, the total cost was reduced by 2.6-fold for proton therapy [70,71]. In a recent report using an influence diagram to model for radiation delivery in lung cancer, the overall costs (radiation plus toxicity costs) and upfront proton treatment costs exceeded that of photons [72]. The relatively lower rate of pneumonitis and esophagitis rates help protons to recover some of the total cost. Peeters et al. [73] described higher costs for the combined proton and lower costs for the photon, and the cost for lung cancer between particle and photon therapies involves a relatively small difference. Grutters et al. [74] analyzed the cost-effectiveness in inoperable stage I NSCLC, costs for quality-adjusted life years (QALYs) of proton therapy, carbon-ion therapy, 3DCRT, and SBRT were 2.33, 2.67, 1.98, and 2.59, respectively, which is the lowest for carbon-ion therapy. Grutters et al. [74] recommended not adopting proton as a standard treatment for NSCLC. Though current costs favor photon therapy in most studies, the preference for proton therapy may be found with relatively small reductions in the cost of proton therapy [72]. Therefore, it is hard to draw a conclusion now, based on the present limited evidence of cost-effectiveness for proton therapy over other therapies in treating lung cancer. More evidence is needed to support evidence-based treatment decisions [74].

## 5. Perspective

In recent years, the high rate of local failure and tumor recurrence in NSCLC was still a hard-hitting issue, though immunotherapy and targeted therapies developed rapidly in the treatment of NSCLC, [12,75]. Proton therapy is expected to further decrease the local tumor recurrence and reduce the toxicities, especially in patients with pulmonary disease/dysfunction. Despite the uncertainty of the proton range of patients with NSCLC, mitigation of the temporal effects, and potential dose discrepancies, proton therapy still needs to be fully integrated. Due to the limitations of this review, which are that the review only included selected studies but not all retrospective studies and did not compare or analyze the quality/limitation of each selected study, there is currently a lack of robust evidence to indicate its clinical superiority. Meanwhile, the cost of proton therapy is higher than that of photon therapy. Therefore, it is expected that the cost-effectiveness will need to be improved before proton therapy is routinely recommended. Furthermore, specific regimens of hypo-fractionated or dose-escalation for proton therapy (especially PBS) are warranted in further studies. 

## 6. Conclusions

Proton therapy is a promising treatment for NSCLC, while direct comparisons of dosimetry, efficacy, and safety, and cost-effectiveness with photon therapy in prospective studies are warranted before proton therapy can be routinely recommended.

## Figures and Tables

**Table 1:** (**A**). Published dosimetric comparative study involving proton therapy (PSPT) for NSCLC; (**B**) Published dosimetric comparative study involving proton therapy (IMPT) for NSCLC (continued).

(A)									
Authors	Design	Year	Cases	NSCLC Stage	Treatment	Dose(Gy)	Fractions	CTV Dosimetric Outcomes (Gy)	OAR Dosimetric Outcomes (Gy)
Wang et al. [13]	-	2009	24	I	PSPT/3D-CRT	66	10	95% isodose line covered 86.4% CTV for proton, and 43.2% for 3D-CRT	Proton delivers lower mean doses to the ipsilateral lung, total lung, heart, esophagus, and spinal cord
Wink et al. [15]	Retrospective	2018	24	I	IMRT/VMAT/CyberKnife/PSPT	60	8	Scattered proton has a lower Dmean of CTV (65.1/65.7/68.1/63.6) and D2% (70.6/70.3/72.9/67.4)	Doses to the spinal cord were lowest with PSPT
Roelofs et al. [17]	Prospective	2012	25	IA-IIIB	3D-CRT/IMRT/PSPT	70	35	-	Higher integral dose for 3D-CRT (59%) and IMRT (43%); Reduced mean lung dose for PSPT (18.9/16.4/13.5, respectively)
Ohno et al. [19]	-	2015	35	3IIB/15IIIA/17 IIIB	Proton/CRT	74	37	45.7% of the X-ray/17.1% of the proton plans were inadequate	Mean lung dose and V5 to V50 were significantly lower in proton
Giaddui et al. [23]	Phase III trial	2016	26	II- IIIB	PSPT/IMRT	70	35	Dose parameters for the target volume were very close for the IMRT and PSPT plans	Lower dose for PSPT plans: lung V5 (34.4 vs. 47.2); maximum spinal cord dose (31.7 vs. 43.5 Gy); heart V5 (19 vs. 47); heart V30 (11 vs. 9); heart V45 (7.8 vs. 12.1); heart V50% (7.1 vs. 9.8) and mean heart dose (7.7 vs. 14.9)
Wu et al. [22]	Retrospective	2016	33	III	PSPT/3D-CRT	60–66	33	-	All the dose parameters of proton therapy, except for the esophageal the dose was lower than 3D-CRT
Shusharina et al. [24]	Retrospective	2018	83	II–IV	IMRT/PSPT	74	37	-	Higher Lung V5 for IMRT, whereas higher V60 for protons; The mean lung dose was similar
**(B)**									
**Authors**	**Design**	**Year**	**Cases**	**NSCLC Stage**	**Treatment**	**Dose(Gy)**	**Fractions**	**CTV Dosimetric Outcomes (Gy)**	**OAR Dosimetric Outcomes (Gy)**
Register et al. [14]	-	2011	15	I	PSPT/IMPT/SBRT	-	-	Only 6 photons, 12 PSPT, and 14 IMPT were satisfied	PSPT and IMPT reduced mean total lung dose from 5.4 to 3.5 and 2.8, and total lung volume receiving 5 Gy, 10 Gy, and 20 Gy
Zhang et al. [16]	-	2010	20	IIIB	IMRT/PSPT/IMPT	74		IMPT prevented lower-dose target coverage in complicated cases	IMPT spared more lung, heart, spinal cord, and esophagus
Berman et al. [18]	Retrospective	2013	10	IIIA	PSPT/IMPT/IMRT	50.4	28	-	IMPT decreases the dose to all OARs. PSPT reduces the low-dose lung bath, increases the volume of lung receiving high dose
Kesarwala et al. [20]	-	2015	20	14IIIA/6IIIB	Proton IFRT/ENI vs. photon IFRT/ENI	66.6–72	36–40	Proton IFRT/ENI both improved D95-PTV coverage by 4% compared to photon IFRT	Decreased lung V20/mean lung dose by 18%/36%, mean esophagus dose by 16% with proton IFRT and by 11%/26%, 12% with proton ENI. Heart V25 decreased 63% with both
Inoue et al. [21]	-	2016	10	III	IMPT/VMAT	60	25	IMPT showed better target homogeneity than VMAT	IMPT reduced 40% mean lung and 60% heart dose
Li et al. [25]	-	2018	14	III	SPArc/IMPT	66	33	Similar robust target volume coverage	SPArc reduced the doses to critical structures as well as the interplay effect
Liu et al. [26]	Retrospective	2018	24	III	VMAT/IMPT	60	-	Comparable CTV dose homogeneity	IMPT with lower cord Dmax, heart Dmean and lung V5 Gy and better robustness in heart Dmean, but worse in CTV dose coverage, cord Dmax, lung Dmean, and V5 Gy
Ferris et al. [27]	Retrospective	2019	26	III	IMPT/VMAT	60	30	-	IMPT improves cardiac dosimetry metrics, maintaining/improving other thoracic OAR constraints

- = not available; NSCLC = non–small-cell lung cancer; CTV = Clinical target volumes; OAR = Organ at risk; IMRT = intensity-modulated radiotherapy; VMAT = volumetric modulated arc therapy; PSPT = passive scattering proton therapy; 3D-CRT = 3-dimensional conformal radiotherapy; IMPT = intensity-modulated proton therapy; IFRT = involved-field radiation therapy; ENI = elective nodal irradiation; SPArc = spot-scanning proton arc therapy.

**Table 2:** Published prospective study/nationwide retrospective study involving proton therapy for early-stage NSCLC.

Authors	Design	Year	Cases	Mean/Median Age (y)	Male (%)	NSCLC Stage	Treatment	Efficacy				Safety
								OS	DFS/PFS	LCR	Others	
Bush et al. [43]	Prospective study	1999	37	72	15	stage I, 27; stage II, 2; stage IIIa, 8	18 combination of protons and x rays/19 proton therapy	-	2-ys DFS 63%	2-ys 87%	-	2 pneumonitis resolved with oral steroids; otherwise, no significant toxicities
Hata et al. [44]	Prospective study	2007	21	74	16	Stage I (T1–2N0M0) NSCLC	Hypo-fractionated proton therapy	2-ys 74%	-	2-ys 95%	2-ys cause-specific survival 86%	No therapy-related toxicity of Grade >3
Bush et al. [45]	Phase 2 study	2013	111	73.2	59	T1 or T2, N0, M0 NSCLC	51,60, and 70 Gy proton therapy	4-ys 18%(51 Gy), 32%(60 Gy), 51%(70 Gy)	-	4-ys 96% (T1)	4-ys OS 60% (70 Gy)	Pneumonitis was not significant and pulmonary function was well maintained
Iwata et al. [46]	Clinical study based on protocols	2013	Proton 43/ Carbon 27	75	51	30 T2aN0M0/13 T2bN0M0	Proton therapy/ Carbon-Ion therapy	4-ys 58%	4-ys PFS 46%	4-ys 75%	4-ys regional recurrence rate 17%	Grade 3 pulmonary toxicity observed in two patients
Chang et al. [47]	Phase I/II prospective study	2017	35	73	16	12 T1/23 T2-3N0M0	PSPT	1-y 85.7%, 2-ys 42.9%, 5-ys 28.1%;	5-ys local/regional/distant DFS 85%/89.2%/54.4%	-	-	Dermatitis (grade 2, 51.4%; grade 3, 2.9%) and radiation pneumonitis (grade 2, 11.4%; grade 3, 2.9%)
Kharod et al. [57]	Phase II prospective study	2020	22	72	13	10 T1/12 T2	Proton therapy	3-ys 81%, 5-ys 49%;	-	3-ys 86%	3-ys cancer-specific survival 100%	1 grade 3 bronchial stricture requiring stent
Ohnishi et al. [48]	Nationwide retrospective study	2020	669	76	486	Stage I NSCLC	PSPT	3-ys 79.5%,	3-ys PFS 64.1%	-	3-ys local PFS 89.8%	Grade 2, 3, 4, and 5 pneumonitis 9.8%, 1.0%, 0%, and 0.7%, respectively. Grade ≥3 dermatitis 0.4%. No Grade 4 or severe adverse events, other than pneumonitis, were observed.

- = not available; PSPT = passive scattering proton therapy; 3D-CRT = 3-dimensional conformal radiotherapy; IMPT = intensity-modulated proton therapy; IMRT = intensity-modulated radiotherapy; NSCLC = non–small-cell lung cancer; OS = overall survival; DFS = disease-free survival; PFS = progression-free survival; LCR = local control rate; y = year; ys = years.

**Table 3:** Published prospective study/nationwide retrospective study involving proton therapy for locally advanced NSCLC.

Authors	Design	Year	Cases	Mean/Median Age (y)	Male (%)	NSCLC Stage	Treatment	Efficacy				Safety
								OS	DFS/PFS	LCR	Others	
Sejpal et al. [49]	Comparative study	2011	62proton/66IMRT/74 3D-CRT	67/62/61	34/40/37	Locally advanced NSCLC	Proton therapy/IMRT/3D-CRT + concurrent chemotherapy	Median 15.2/17.4/17.9 months	-	-	-	Rates of severe (grade 3) pneumonitis and esophagitis in the proton group (2% and 5%) were lower (3D-CRT, 30%, and 18%; IMRT, 9%, and 44%)
Nguyen et al. [50]	Prospective study	2015	134	69	73	21 stage II, 113 stage III	PSPT	Median 40.4 (II), 30.4 (III) months	5-ys DFS 17.3% (II), 18.0% (III)	-	-	1 grade 4 esophagitis and 16 grade 3 events (2 pneumonitis, 6 esophagitis, 8 dermatitis)
Hoppe et al. [51]	Phase 2 study	2016	14	65	9	stage IIIA, 9; stage IIIB, 5	Proton therapy delivering 74 to 80 Gy with concurrent chemotherapy	2-ys 57%; Median 33 months	2-ys PFS 25%; Median 14 months	-	-	No acute grade 3 toxicities related to proton therapy. Late grade 3 gastrointestinal 1 and pulmonary toxicity 1
Higgins et al. [52]	Nationwide retrospective	2017	309proton/1549non-proton	68	57% were males	Stage II and III (60%)	Proton vs. photon radiotherapy	5-ys 22% vs. 16%	-	-	-	-
Chang et al. [55]	Phase 2 Study	2017	64	42	70	30 stage IIIA; 34 stage IIIB	PSPT with chemotherapy	Median 26.5 months (5-ys 29%)	5-ys PFS 22%	5-ys 72%	5-ys distant metastasis 54%	Rates of grade 2/3 acute esophagitis 28%/8%. Acute grade 2 pneumonitis 2%. Late toxic effects were uncommon
Liao et al. [53]	Randomized trial	2018	92IMRT/57PSPT	66	80	IIB/IIIB/IV	IMRT/PSPT (both with chemotherapy)	-	-	-	Local failure 10.9% vs. 10.5%	Grade ≥ 3 radiation pneumonitis (IMRT, 6.5%; PSPT, 10.5%)
Elhammali et al. [54]	Prospective study	2019	51	70	29	Advanced NSCLC	Concurrent chemotherapy and IMPT	Median 33.9 months	Median DFS 12.6 months	3-ys 78.3%	-	Grade 3 toxicity rate 18%; grade 2 esophagitis 43%, dermatitis 31%, fatigue 27%
Hoppe et al. [56]	Multicenter phase 1 trial	2020	18	74	16	Stage II or III NSCLC	Chemotherapy with increasing dose-per-fraction proton therapy	-	-	-	-	No severe adverse event related to radiation therapy.

- = not available; PSPT = passive scattering proton therapy; 3D-CRT = 3-dimensional conformal radiotherapy; IMPT = intensity-modulated proton therapy; IMRT = intensity-modulated radiotherapy; NSCLC = non–small-cell lung cancer; OS = overall survival; DFS = disease-free survival; PFS = progression-free survival; LCR = local control rate; y = year; ys = years.
